# Hardness Prediction of MoNbTaW Alloy Films Based on Machine Learning and Interpretability Analysis

**DOI:** 10.3390/ma19030543

**Published:** 2026-01-29

**Authors:** Yan-Han Yang, Tian-You Zhu, Wei Ren, Wei-Li Wang

**Affiliations:** 1 School of Science, Xi’an University of Posts & Telecommunications, Xi’an 710121, China; yangyanhan@xupt.edu.cn (Y.-H.Y.); zhutianyou@stu.xupt.edu.cn (T.-Y.Z.); 2 School of Physical Science and Technology, Northwestern Polytechnical University, Xi’an 710072, China; wlwang@nwpu.edu.cn

**Keywords:** high-entropy alloy, machine learning, hardness prediction, feature selection, ridge regression

## Abstract

Machine learning (ML) offers a powerful paradigm for accelerating performance prediction of high-entropy alloys (HEAs). The present study proposed an ML framework based on the ridge regression algorithm for predicting the hardness of MoNbTaW HEA films. By comparing various feature-screening strategies, an optimized feature set comprising three features, namely *δG*, *Λ*, and *Ω*, was selected from 20 candidate physical features. The model based on this feature set exhibited strong predictive performance. In 10-fold cross-validation, *R*^2^ was 0.86, *RMSE* was 0.41 GPa and MAE was 0.31 GPa. On the reserved validation set, *R*^2^ was 0.88, *RMSE* was 0.37 GPa, and MAE was 0.31 GPa. The model further revealed the influence trends of constituent elements and key features on hardness. By using ML to mine useful information from a dataset of HEA film samples prepared via magnetron sputtering, this work provides an approach for rapid and cost-effective design of HEAs.

## 1. Introduction

In 2004, Yeh et al. [1] postulated that short-range, highly disordered alloys can be obtained by mixing five or more principal elements in equal molar ratios. This type of alloy is classified as a high entropy alloy (HEA). Due to high mixing entropy, HEAs form simple body-centered cubic or face-centered cubic solid solutions. Their essential characteristics include a high entropy effect, lattice distortion effect, sluggish diffusion effect, and cocktail effect [2,3]. The confluence of these effects provides substantial potential for HEAs in terms of mechanical properties, corrosion resistance, and high-temperature stability [4,5]. As researchers have gained deeper understanding of HEAs, it has been recognized that alloys containing four metallic elements can also be classified as HEAs, and the mixing of principal elements does not necessarily follow the requirement of equal molar ratios [6]. HEAs composed mainly of four or more refractory metal elements are known as refractory high-entropy alloys (RHEAs). RHEAs exhibit distinct high-temperature thermomechanical properties, making them one of ideal material groups for applications in extreme high-temperature environments [7]. For instance, NbMoTaW maintains favorable structural stability and high hardness at 1400 °C [8].

It is well known that the properties of materials are influenced by their chemical composition, microstructure, and preparation process. Most alloys possess very complex microstructures and require complicated preparation processes. In comparison, the relationship between the chemical composition and properties of alloys is relatively easy to understand. Therefore, deciding chemical composition is usually the first step in designing HEAs with a desired performance [9]. In theory, HEAs offer unlimited principal element ratios, so it is extremely difficult to quickly sift out HEAs with desired performance. Some commonly used simulation methods, such as thermodynamic simulations, density functional theory (DFT), and molecular dynamics (MDs) simulations can accelerate the discovery of new materials [10,11]. However, when researchers use these methods to calculate the relationship between the composition and performance of HEAs, they often encounter challenges such as high computational complexity, a long development cycle, and low efficiency. With the development of artificial intelligence in recent years, ML based on material computing data and experimental or production data has been widely applied in the materials research [12,13,14]. ML is defined as a data-driven computing approach that builds models by identifying hidden correlations and patterns in large volumes of data [15,16]. It can predict the performance of HEAs, reveal strengthening mechanisms of HEAs, and provide guidance for designing HEAs [17]. Xu et al. [18] have revealed the rugged energy landscape mechanism of the slow diffusion effect in HEAs using a method combining ML and computational simulation. Zhang et al. [19] have used ML to sift out appropriate compositions from a vast array of candidate compositions, and successfully prepared a new breed of high-strength Al alloys. Wen et al. [20] have applied ML to design compositions of RHEAs and have obtained several RHEAs with excellent strength and ductility. Chen et al. [21] have employed a stacked ensemble learning algorithm to design AlNbTiVZr lightweight HEAs with high hardness.

In materials genetic engineering, parallel material preparation and high-speed testing are achieved through application of high-throughput preparation and characterization techniques, greatly shortening experimental cycles and making it possible to prepare a large number of samples in a short time [22]. Composition-property mapping can be established in a large composition space by using ML to mine data from huge numbers of samples obtained using high-throughput preparation techniques [23]. On this basis, relevant properties of materials can be predicted with high efficiency, thus accelerating the speed of discovering new materials [24]. Zhong et al. [25] have developed a chemical reaction prediction model on the basis of crunching data from high-throughput experiments using Bayesian deep learning, achieving accuracy of 89.48% on a test set. Huang et al. [26] have used ML and high-throughput preparation technology to design a (TiZrNbCrSi)N high-entropy nitride coating with hardness of 25.6 GPa and toughness of 8 MPa·m^1/2^.

For the performance prediction of HEAs, current research works predominantly focus on bulk materials. Machine learning models rely heavily on large datasets. And the data from bulk HEAs prepared via traditional methods such as casting, forging, and powder metallurgy are more readily collected and integrated systematically. In contrast, the researchers’ attention to the data from thin-film systems remains relatively limited. Furthermore, it is challenging to achieve an effective balance between the predictive accuracy of the models and their physical interpretability, particularly for RHEAs that suffer from limited data accumulation. In the present study, a hardness prediction framework based on ML was developed using a dataset obtained from MoNbTaW HEA thin films prepared via magnetron sputtering. Based on the comparison of five feature selection methods, a small number of features with clear physical meaning were selected from an initial feature pool containing 20 physical descriptors to form an optimized feature set. Furthermore, the Pearson correlation coefficient (PCC) and SHapley Additive exPlanations (SHAP) methods were used to analyze the mechanisms of key features contributing to hardness, linking data-driven prediction to the physical mechanism of solid-solution strengthening. The purpose was to combine high-throughput preparation with machine learning to provide a method for the rapid and cost-effective design of HEAs.

## 2. Theory and Methodology

### 2.1. Model Design Strategy

**Figure 1:**
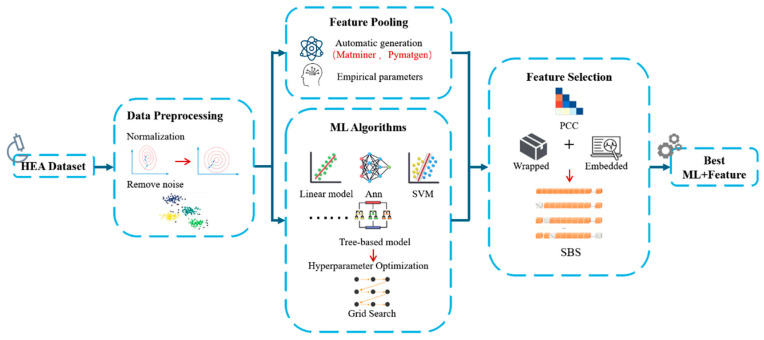
Flowchart of predicting the hardness of RHEAs thin films.

The objective of the present study was to construct a high-performance hardness prediction model for HEA films. As shown in Figure 1, the research process included five key steps: data preparation and feature engineering, initial selection and evaluation of ML algorithms, multi-strategy feature screening, model training and interpretability analysis, and model validation and performance evaluation. All calculations and analyses were conducted on programs written in the Python programming language (version 3.12.7), and ML libraries used in this study included Scikit-Learn version 1.5.1, Numpy version 2.0.2, Pandas version 2.2.2 and XGBoost version 2.1.4, while data visualization was performed using Matplotlib version 3.9.2 and Seaborn version 0.13.2.

### 2.2. Data Source and Preprocessing

The dataset used in the present study was compiled based on the dataset of Ref. [27], which has been published on the Zenodo data platform. It constituted a small-to-medium-sized dataset containing composition and hardness data of MoNbTaW quaternary high-entropy alloy films prepared by magnetron sputtering, comprising a total of 311 sample groups, and the composition ranges of principal elements were: Mo (9–31 wt%), Nb (20–59 wt%), Ta (10–42 wt%), and W (12–32 wt%). When input variables are scaled to a standard range of values, many machine learning models tend to perform better [13]. To achieve this, datasets are typically normalized using either standardization or normalization. Given that min–max normalization deterministically maps all features to a uniform [0, 1] interval, it provides a fair benchmark for subsequent analysis of feature contributions based on distance or weight. Therefore, this study adopted this method to scale all features in order to eliminate the influence of dimensional units. The *max*–*min* normalization formula was as follows [21]:

(1) $$x^{\prime }=\left(x-\min\left(X\right)\right)/\left(\max\left(X\right)-\min\left(X\right)\right)$$

**Figure 2:**
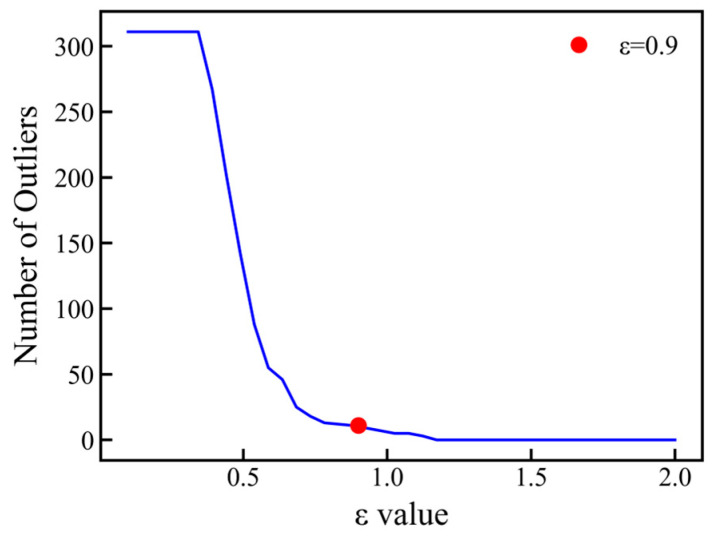
Sorted k-distance graph for determining the neighborhood radius ε of the DBSCAN algorithm with MinPts = 7.

**Figure 3:**
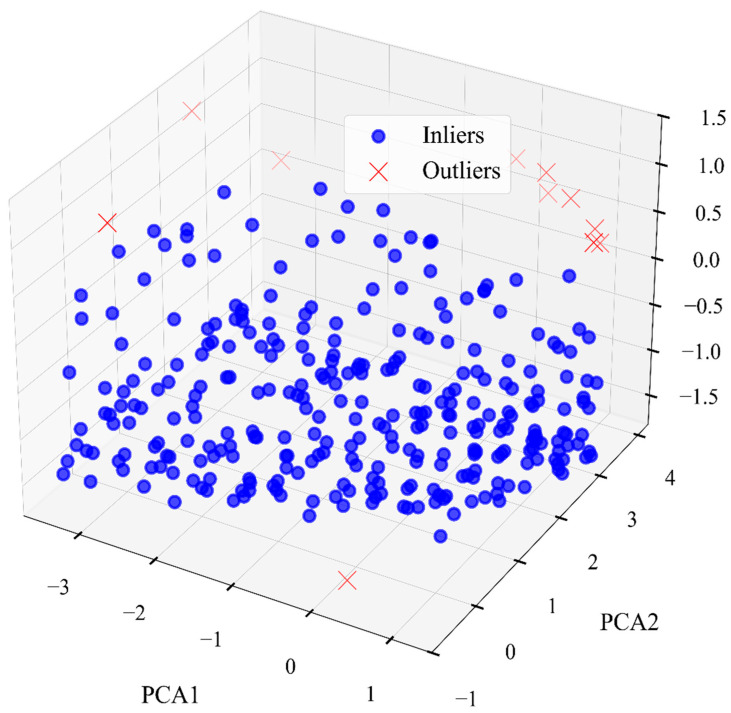
Visualization result of noise removal using DBSCAN.

Outliers in the dataset were detected and removed using the density-based spatial clustering of applications with the noise (DBSCAN) algorithm. This algorithm could effectively identify noise points in clusters of any shapes, demonstrating strong robustness in identifying potential outliers in datasets. In the process of using DBSCAN to remove outlier data points from small or clean datasets, one can start from the empirical guideline of MinPts ≥ D + 1, or choose a more robust default of MinPts ≥ 2 × D. Since the initial features of the dataset consisted of the compositions of four elements and their hardness, MinPts was set to 7. After fixing MinPts, the neighborhood radius ε = 0.9 was determined by plotting the sorted k-distance graph (Figure 2) and identifying its “elbow point.” The results are shown in Figure 3. A comparison between using and not using DBSCAN is made in Section 3.3. To ensure the reliability of model evaluation, 90% of the dataset was used as the main training–validation set, which was employed in the 10-fold cross-validation process for model tuning; the remaining 10% of the data served as an independent test set and was not involved in model training. It should be clearly stated that the prediction model developed in this study is specifically tailored for the MoNbTaW refractory high-entropy alloy system prepared via magnetron sputtering. While this work provides a focused and effective framework for this specific material system, its generalizability to other RHEA compositions or material preparation routes is inherently limited by the scope of the training data. External validation using independent datasets from different RHEA systems would be highly valuable for assessing its potential transferability. However, due to the scarcity and high cost of standardized experimental hardness data for RHEA thin films, such validation has not yet been completed. Given this data limitation, and in line with similar studies [28], priority has been given to constructing and optimizing a model for the specific system.

### 2.3. Feature Engineering and ML Algorithm

Features are input parameters of a model, which carry information in data and, to a large extent, determine performance, interpretability, and generalization ability of the model. Use of high-quality features is conducive to capturing patterns in data effectively, thereby improving the performance of a model. Based on theories and literature in materials science [29,30], an initial feature set was constructed containing 20 physical descriptors to characterize atomic structure, electronic properties, and thermodynamic properties of HEAs. These features included mixing enthalpy (Δ*H*_mix_), mixing entropy (Δ*S*_mix_), atomic size difference (*δr*), valence electron concentration (*VEC*), electronegativity difference (Δ*χ*), and modulus difference (Δ*G*, *δE*), as well as empirical parameters *γ* and *Ω* [31], Peierls–Nabarro factor [32], etc. (Table 1). The initial feature set primarily comprised physical descriptors related to alloy composition and thermodynamic properties, designed to capture the influence of composition-dominated solid solution strengthening on thin-film hardness. However, film hardness is also significantly controlled by microstructural factors—such as grain size, texture, and residual stress—which are closely tied to the preparation process. Given that the dataset used in this study was obtained from samples prepared under the same magnetron sputtering conditions, and the current feature set focuses on compositional descriptors, the model primarily reveals the dominant trend of hardness variation with composition within a relatively fixed processing window.

Choice of ML algorithms is equally crucial for building prediction models. Although some existing algorithm libraries can meet the requirements of many tasks such as classification, regression, and clustering, there is no universal algorithm that can achieve optimal performance on all datasets. Linear models such as ridge regression and Lasso offer high interpretability and stability when capturing linear trends, but they struggle to directly model complex nonlinear relationships. Support Vector Regression can handle nonlinear relationships through kernel functions, but typically requires careful parameter tuning, suffers from lower training efficiency on large-scale datasets, and generally provides less interpretability compared to linear models. Ensemble methods such as Random Forest and Gradient Boosting are capable of effectively modeling complex feature interactions, yet they are prone to overfitting and often have reduced interpretability. Simpler models such as Decision Trees are computationally efficient and easily interpretable, but may lack predictive robustness. Although the K-Nearest Neighbors algorithm is conceptually straightforward, its prediction cost becomes high for large datasets. Therefore, carefully screening and optimizing algorithms based on data characteristics of specific material systems (including sample size, feature dimension, and noise distribution) and intrinsic physicochemical relationships is essential for improving generalization ability and robustness of models. This process not only improves prediction accuracy, but also ensures that computational frameworks can effectively capture key descriptors that influence the performance of materials, thereby providing reliable theoretical guidance for design and discovery of new materials. To comprehensively evaluate the performance of models, a variety of representative ML algorithms were selected for comparison, including traditional regression algorithms such as ridge regression, Lasso Regression, Support Vector Regression, K-Nearest Neighbors, and Decision Tree, and ensemble learning algorithms such as Random Forest, Extreme Gradient Boosting (XGBoost), and Gradient Boosting Decision Tree, as well as neural networks. All models were trained and evaluated using the 10-fold cross-validation method to make full use of limited data and reduce evaluation biases caused by data splitting. The dataset was evenly divided into ten subsets. In each iteration, nine subsets were used as training sets and the remaining subset as the test set. Finally, average values obtained in ten validation rounds were taken as values of model performance indicators. In this study, MAE, RMSE, and R^2^ from the Scikit-Learn library were used to evaluate predictive performance [16], thereby providing a comprehensive assessment of the model’s accuracy, error magnitude, and explanatory power in describing the variability in the data. These metrics are defined as follows:

(2) $$MAE=\frac{1}{n}\sum_{i=1}^{n}\left|\hat{y_{i}}-y_{i}\right|$$

(3) $$\mathrm{RMSE}=\sqrt{\frac{1}{n}\sum_{i=1}^{n}{\left(y_{i}-\hat{y_{i}}\right)}^{2}}$$

(4) $$R^{2}=1-\frac{\sum_{i=1}^{n}{\left(y_{i}-\hat{y_{i}}\right)}^{2}}{\sum_{i=1}^{n}{\left(y_{i}-\overset{-}{y}\right)}^{2}}$$

where n represents sample size, $y_{i}$ denotes true value, and $\hat{y_{i}}$ stands for predicted value, where $\overset{-}{y}$ is average value of true value. The selection of these three metrics is based on their complementary roles in regression evaluation. Considering that the hardness values in this dataset (approximately 4–10 GPa) represent a relatively concentrated range that does not span multiple orders of magnitude, MAE and RMSE are appropriate for capturing prediction errors. R^2^ reflects the proportion of variance explained by the model, making it suitable for assessing the overall goodness of fit; RMSE amplifies larger errors by squaring the residuals, rendering it sensitive to outliers; while MAE provides a more intuitive and robust measure of average error magnitude, being less influenced by extreme values.

### 2.4. Feature Screening

When the number of features in a feature pool is too large, it leads to increases in model complexity, risk of overfitting, and consumption of computing resources. Moreover, the model may allocate overly heavy weights to weakly-correlated features, resulting in weaker generalization ability of the model. To optimize model performance, address the issue of high-dimensional data redundancy, and enhance model interpretability, the PCC method was used to analyze the correlation between features, and five feature selection methods were compared to identify the most predictive physical descriptors. The PCC is defined as the quotient of the covariance and the standard deviation of two variables, which is used to evaluate the linear correlation between the two variables. The formula for calculating the PCC is as follows [14]:

(5) $$PCC=\frac{\sum_{i=1}^{n}\left(X_{i}-\overset{-}{X}\right)\left(Y_{i}-\overset{-}{Y}\right)}{\sqrt{\sum_{i=1}^{n}{\left(X_{i}-\overset{-}{X}\right)}^{2}}\sqrt{\sum_{i=1}^{n}{\left(Y_{i}-\overset{-}{Y}\right)}^{2}}}$$

where $X_{i}$ and $Y_{i}$ represent two types of features, and X and Y stand for mean values of $X_{i}$ and $Y_{i}$, respectively. When the PCC is lower than 0, it indicates a negative correlation. A value higher than 0.9 indicates a strong correlation.

The compared feature selection methods included the following: (1) Recursive feature elimination (RFE), which was the core algorithm of the wrapping method. Starting from the full feature set, RFE recursively eliminated redundant features based on the feature importance ranking of the model algorithm. In each round of iteration, performance of the feature subset was evaluated using the 10-fold cross-validation method with *RMSE* serving as the metric, retaining optimal feature combination. (2) Sequence forward selection (SFS) and sequence backward selection (SBS), both of which belonged to category of wrapping method. SFS gradually added features that could improve model performance to the largest extent, starting from empty feature set. SBS gradually removed features that could degrade model performance to the largest extent, starting from full feature set. Both methods used cross-validation *RMSE* as the criterion for feature addition/deletion. (3) The genetic algorithm (GA). The GA simulated natural selection mechanisms (selection, crossover, and mutation) through binary coding, and used the reciprocal of *RMSE* as the fitness function to globally search for the optimal feature subset under specific settings (population size: 30, number of evolution generations: 20). (4) Embedding. The idea of the embedding method was to embed feature selection within the model training process, and use feature importance weights obtained from model training for feature screening.

## 3. Results and Discussion

### 3.1. Optimal ML Algorithm

In the present study, ML algorithms were trained in sequence, with 20 candidate material features taken as input parameters and the HEAs’ hardness value as output value. To give full play to the advantages of each ML algorithm, the hyperparameters corresponding to minimum *RMSE* value were searched out using the grid search method in conjunction with the 10-fold cross validation method in course of algorithm training.

**Figure 4:**
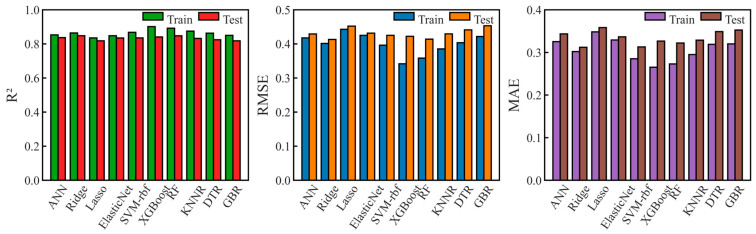
Fitting results of 10 ML algorithms obtained on compiled dataset.

Figure 4 shows the performance comparison of the compared ML algorithms. As can be found by observing *R*^2^ values, the ridge regression algorithm achieved the best performance on the dataset compiled for the present study, yielding a cross-validation *R*^2^ value of 0.848 and an *RMSE* value of 0.414 (see Table 2). This indicated that ridge regression had a better fitting effect and prediction ability when dealing with compiled dataset. Although ensemble learning algorithms such as RF and XGBoost also performed well, the differences between the results obtained on the training sets and the test set indicated a certain degree of overfitting. Considering the stability of ridge regression and its potential in model interpretation, it was adopted as the benchmark algorithm to screen the features.

### 3.2. Feature Screening and Interpretation

**Figure 5:**
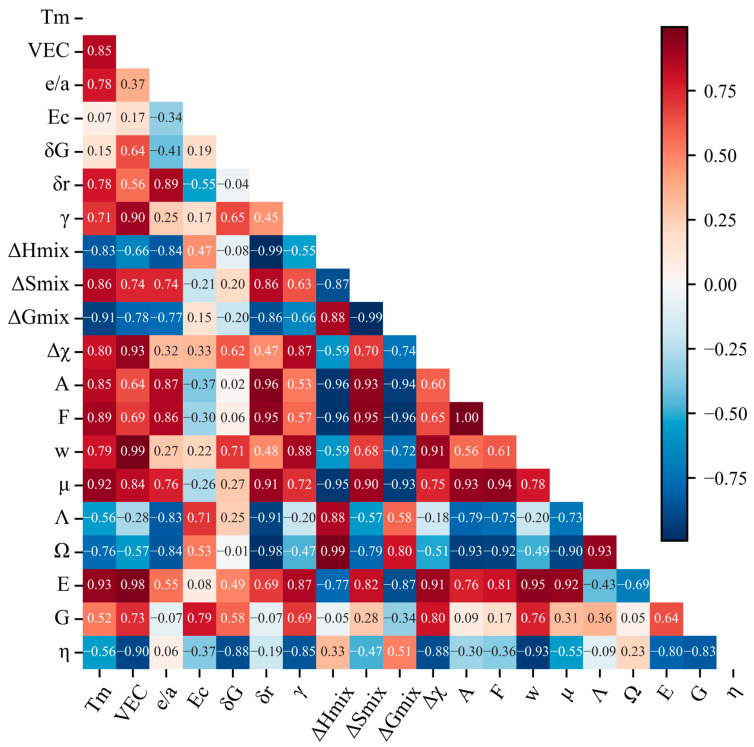
PCC heat map with added features.

**Figure 6:**
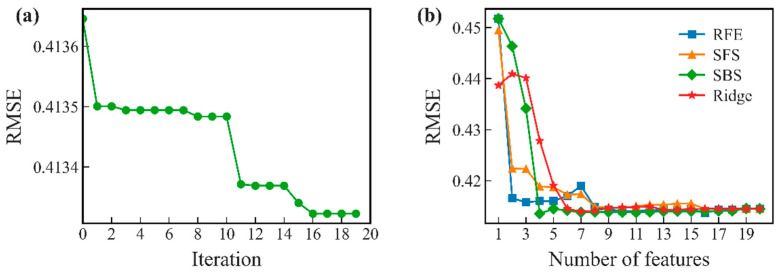
(**a**) Iterative curve of feature search using genetic algorithm and (**b**) RMSE values of SBS, SFS, RFE, and ridge under different eigen numbers.

Figure 5 shows PCC values between features and HEA hardness. There are multiple feature combinations with a correlation degree greater than 0.9 shown in the figure. For these feature combinations, deleting unimportant features through feature importance assessment could lead to the problem of neglecting the synergistic promoting effect of multiple features on HEA hardness. Although an exhaustive method could traverse all feature combinations and find the global optimal feature subset, the process of feature screening became extremely slow when the number of candidate features was large. This occurred because computational complexity of the exhaustive method increased exponentially with increase in the number of features. The ridge regression model was adopted to compare several feature selection methods. As shown in Figure 6b, the RMSE of the features selected by RFE, SFS, and SBS, as well as by the wrapping method and the embedding method based on ridge regression tended to stabilize within eight features. The best performance was achieved when retaining 16, 16, 4, and 7 features (see Table 3), respectively. Figure 6a shows the iteration plot of the GA, which yielded better results than other feature selection methods. After 16 iterations of global search, GA yielded the eight-element optimized feature combination [*VEC*, *δG*, *δr*, *γ*, *ΔH*_mix_, *ΔG*_mix_, *F*, *G*]. The four-element feature set [*δG*, *Λ*, *Ω*, *G*] selected by SBS had a relatively low *RMSE* value. To further examine the feature set and enhance its interpretability, the contribution of each feature was analyzed. It was found that when only three core features *δG*, *Λ*, and *Ω* were used, model performance tended to stabilize, and its *RMSE* value was comparable to that of the four-feature model, indicating that marginal contribution of the feature G was limited. Therefore, *δG*, *Λ*, and *Ω* were eventually selected as the optimized feature set of the ridge regression model, which reduced the complexity of the model.

ML models are often deemed as “black boxes” whose inner working principles are not clear. Overcoming the “black box” nature of machine learning models to establish transparent and trustworthy predictive frameworks has become a key research direction in materials science. Current mainstream approaches to model interpretability primarily fall into two categories: “Post hoc explanation,” which aims to understand the knowledge already learned by the model—such as revealing the influence of input variables (e.g., electronegativity and reaction activity of elements) on prediction outcomes through feature importance analysis—and “Transparency,” which focuses on the internal workings of the model, constructing a clear structure from the outset of model design and examining how the model overall processes and propagates information. Embedding domain knowledge into feature engineering or utilizing interpretable mathematical representations to construct input features can enhance the physical interpretability of the model from its source. Furthermore, as a single interpretability method may have limitations, adopting multi-faceted, interactive explanatory model analysis (IEMA) can provide a more comprehensive and reliable understanding. The SHAP model, first proposed by Lundberg and Lee in 2016 [33], could further explain the relationship between features and target values. SHAP is a game theory approach that explains the output of an ML model by reflecting the importance of each feature in every sample. When using SHAP to analyze an ML model, not only could the global importance of individual features be obtained, but the global influence of individual sample features on the target value could also be obtained. This allowed for a global interpretation of the ML model. The SHAP value is described as follows [33]:

(6) $$Y_{i}=Y_{base}+\sum_{i=1}^{k}f\left(X_{i_{k}}\right)$$

where the i-th sample is $X_{i}$, the k-th feature of i-th sample is $X_{i_{k}}$, and the model-predicted value of this sample is $Y_{i},$ i.e., hardness. $Y_{base}$ represents the average predicted value of the model, while $f(X_{i_{k}})$ is the SHAP value of the k-th feature of the sample i. When f(X) > 0, it indicates that this feature enhances predicted value; when f(X) < 0, it indicates that this feature weakens predicted value.

**Figure 7:**
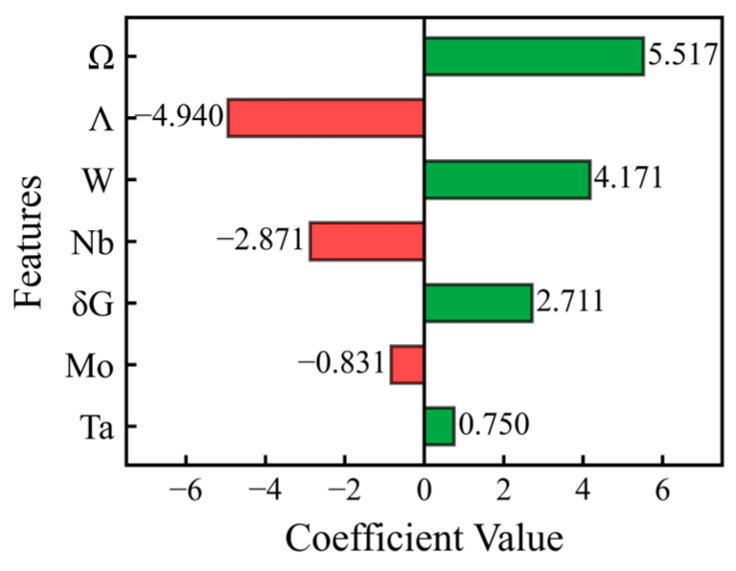
Ridge regression coefficient values of three optimized features selected by model and four principal elements.

**Figure 8:**
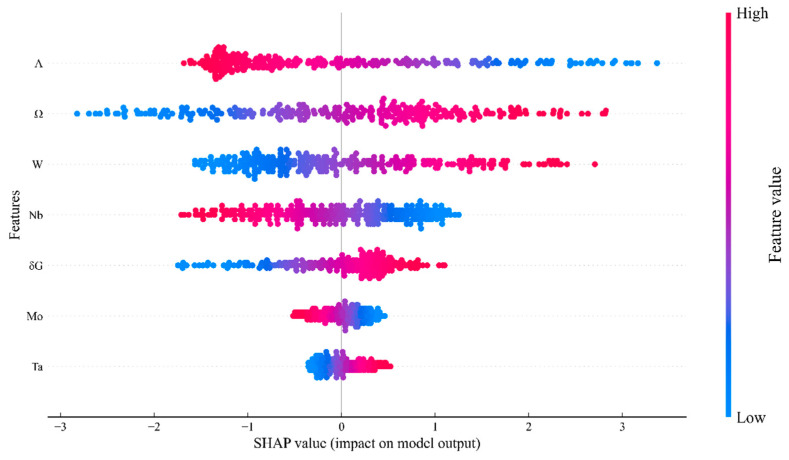
SHAP analysis results of three optimized features selected by the model and four principal elements. The sign of the SHAP value of each scattered point reflects the strengthening or weakening effect of the value of feature on the hardness of the current sample point.

However, the ML model was not a complete “black box”. Figure 7 and Figure 8 show the interpretations of the physical correlation between physical descriptors selected by the ridge regression model and the hardness of MoNbTaW HEAs, as explained by the ridge regression model coefficients and the SHAP results, respectively. The results from both methods demonstrated a high degree of consistency. This way, model-made decisions could be explained as follows: *Ω* parameter, which described the ability of solid-solution formation, was identified as most important positive descriptor. A larger *Ω* value meant that the entropy effect was more dominant and more conducive to the formation of a random and disordered solid-solution phase. This indicated that stable single-phase solid solution structure was a prerequisite for achieving high hardness. Thirathipviwat et al. [9] have found that the increase in microhardness of HEAs is positively correlated with *δr*, which can be largely attributed to high lattice distortion. The strong negative influence of the Λ parameter ΔSmix/δr indicated that although *δr* strengthened through lattice distortion, an excessively high entropy-to-size ratio was not conducive to hardness optimization. This indicates that under the premise of maintaining the stability of a solid solution, moderate lattice distortion was more conducive to the improvement of mechanical properties compared with extreme disorder. W and Ta were key strengthening elements, while Nb should be restricted in use or replaced by other elements. This model quantitatively captured and isolated the contributions of multiple solid-solution strengthening mechanisms. This provides a data-driven perspective for understanding the hardness strengthening in the MoNbTaW system, indicating that the model aims to identify design-relevant compositional trends through effective descriptors, without delving into the elucidation of fundamental mechanisms.

### 3.3. Model Prediction Results and Discussion

**Figure 9:**
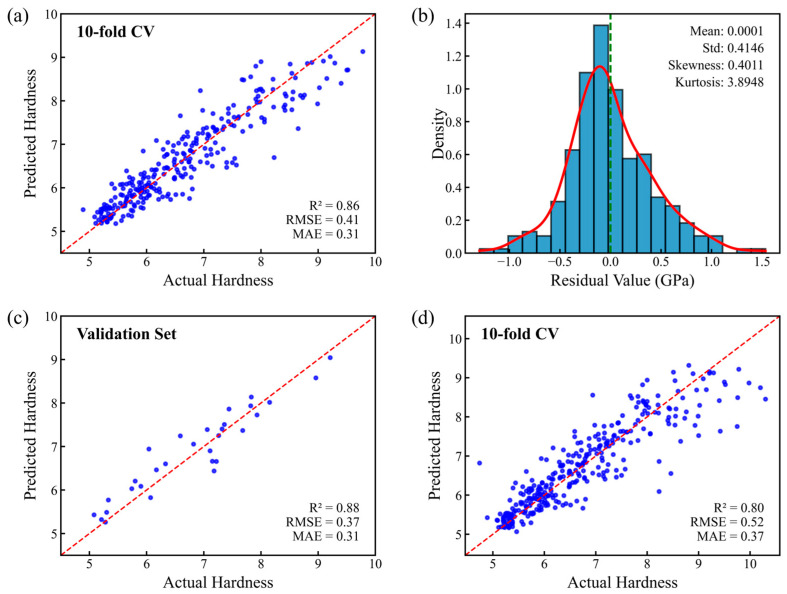
(**a**) Model fitting results under 10-fold cross-validation using the optimized feature set. (**b**) Statistical analysis of the residual distribution for predictions from the 10-fold cross-validation. (**c**) Model fitting results on the reserved independent validation set. (**d**) Model fitting results under 10-fold cross-validation without DBSCAN-based outlier removal.

As shown in Figure 9a, the 10-fold cross-validation results demonstrate stable model performance (R^2^ = 0.86, RMSE = 0.41 GPa, MAE = 0.41 GPa), indicating good robustness to different data splits. Figure 9c shows that after verification on the reserved validation set, the model achieved strong performance (*R*^2^ = 0.88, *RMSE* = 0.37 GPa, MAE = 0.31 GPa), indicating that it could accurately predict unknown components in the same dataset. The residual analysis results from the ten-fold cross-validation (Figure 9b) show that the mean residual is close to zero (0.0001), indicating that the model is generally unbiased. The skewness is 0.4011, reflecting a mildly right-skewed distribution, which implies the presence of a small number of samples whose actual hardness values are higher than the predicted values. This may be attributed to the relatively lower proportion of high-hardness samples in the dataset. The kurtosis is 3.8948, suggesting that the residual distribution has slightly heavier tails compared to a normal distribution, yet no significant abnormal errors are observed. Overall, the characteristics of the residual distribution are reasonable, reflecting good stability in the model predictions. To evaluate the impact of outlier removal on model performance, a comparison was made between the results with and without DBSCAN processing. As shown in Figure 9d, after removing outliers using DBSCAN, both the R^2^ and RMSE of the model showed certain improvements, indicating that this preprocessing step helps enhance the predictive accuracy of the model. This improvement mainly stems from DBSCAN’s ability to identify and exclude samples located in minority classes or sparse data regions, thereby allowing the model to better capture the main structure of the data while preserving the majority of representative samples. However, it should also be noted that this method, while eliminating outliers, may also exclude some samples lying at the distribution boundaries, which themselves may lack sufficient representativeness. Therefore, if additional experimental data are introduced in future studies, previously removed samples should be reincorporated, and outlier detection and screening should be performed again based on the updated complete dataset. This is because some samples initially labeled as “abnormal” may be due to the limited statistical representativeness resulting from the small sample size of the original dataset, rather than being genuine anomalies. It should be noted that nanoindentation hardness measurements inherently carry experimental uncertainty, the magnitude of which varies with material systems and testing conditions. Even under strict calibration, the standard uncertainty of hardness measurement for the standard reference material tungsten is about 0.5 GPa [34]; for amorphous alloys, the standard deviation of hardness measurements obtained using an optimized data-analysis workflow lies in the range of 0.42–0.56 GPa [35]. The prediction error of our model just falls into this range. Inherent measurement uncertainty also serves as a primary source of the model’s error.

However, the model of the present study was tested mainly on hardness data of a batch of quaternary MoNbTaW HEA samples prepared using the same magnetron sputtering process. The universality of this model framework needs to be further verified in following way: apply the model to other refractory HEA systems such as VNbTaMoW to prepare samples using different preparation processes, and use hardness data of these samples to conduct multiple rounds of model training and testing. In addition, the current feature set mainly consisted of thermodynamic and structural parameters. This means that the influence of microstructural features such as the grain-boundary strengthening effect and process parameters such as sputtering power and substrate temperature on hardness have not been considered sufficiently. In future research, it will be necessary to quantify the uncertainty of model predictions to assess their reliability, and features related to grain-boundary structure, process conditions, etc., will be introduced to reveal key factors affecting hardness more comprehensively to enhance the interpretability and generalization ability of the model.

## 4. Conclusions

The present study proposed a framework for predicting the hardness of RHEA films, which encompassed data preprocessing, ML algorithm selection, feature screening, and model verification and interpretation. This framework was helpful in the effort to design RHEA films with higher hardness. By using a ridge regression model established based on the optimized feature set containing δG, Λ, and Ω, the model yielded R^2^ of 0.86 and RMSE of 0.41 on a compiled dataset under 10-fold cross-validation. SHAP analysis and model coefficients indicated that Ω and δG had a positive impact on hardness. For the MoNbTaW system, increasing W content and reducing Nb content would help improve the hardness of HEA thin film. This method may provide guidance on how to mine high-throughput experimental data of HEAs using ML.

## Figures and Tables

**Table 1:** Twenty empirical feature parameters related to hardness of HEAs and their formulas.

$T_{m}$	$\sum_{i=1}^{n}c_{i}T_{m,i}$	δr	$\sqrt{\sum_{i=1}^{n}c_{i}{\left(1-r_{i}/\bar{r}\right)}^{2}}$	F	2G/(1−μ)
VEC	$\sum_{i=1}^{n}c_{i}VEC_{i}$	γ	$\frac{1-\sqrt{\left[{\left(\bar{r}+r_{min}\right)}^{2}-\bar{r^{2}}\right]/{\left(\bar{r}+r_{min}\right)}^{2}}}{1-\sqrt{\left[{\left(\bar{r}+r_{max}\right)}^{2}-\bar{r^{2}}\right]/{\left(\bar{r}+r_{min}\right)}^{2}}}$	w	${\left(\sum_{i=1}^{n}c_{i}w_{i}\right)}^{6}$
e/a	$\sum_{i=1}^{n}c_{i}{\left(e/a\right)}_{i}$	ΔHmix	$\sum_{i\neq j}c_{i}c_{j}\Delta H_{ij}$	μ	0.5Eδr
E	$\sum_{i=1}^{n}c_{i}E_{i}$	ΔSmix	$-R\sum_{i=1}^{n}c_{i}\ln c_{i}$	Λ	$\Delta S_{\mathrm{mix}}/\delta r$
G	$\sum_{i=1}^{n}c_{i}G_{i}$	ΔGmix	$\Delta H_{\mathrm{mix}}-T_{m}\Delta S_{\mathrm{mix}}$	Ω	$T_{m}\Delta S_{\mathrm{mix}}/\left|\Delta H_{\mathrm{mix}}\right|$
Ec	$\sum_{i=1}^{n}c_{i}E_{c,i}$	Δχ	$\sqrt{\sum_{i=1}^{n}c_{i}{\left(\chi_{i}-\bar{\chi }\right)}^{2}}$	η	$\frac{\sum_{i=1}^{n}c_{i}\left(2\left(G_{i}-G\right)/\left(G_{i}+G\right)\right)}{\sum_{i=1}^{n}c_{i}\left(1+0.5\left|2\left(G_{i}-G\right)/\left(G_{i}+G\right)\right|\right)}$
δG	$\sqrt{\sum_{i=1}^{n}c_{i}{\left(1-G_{i}/G\right)}^{2}}$	A	Gδr(1+μ)/(1−μ)		

**Table 2:** Performance evaluation results of 10 machine learning models on the test set of the compiled dataset.

Algorithm	Ann	Ridge	Lasso	ElasticNet	SVM-rbf	XGBoost	RF	KNNR	DTR	GBR
R^2^ (test)	0.8368	0.8481	0.8183	0.8350	0.8352	0.8406	0.8475	0.8320	0.8247	0.8185
RMSE (test)	0.4293	0.4133	0.4523	0.4316	0.4252	0.4221	0.4139	0.4294	0.4411	0.4531
MAE (test)	0.3436	0.3121	0.3583	0.3366	0.3131	0.3266	0.3222	0.3289	0.3489	0.3526

**Table 3:** Optimized feature sets yielded by different feature selection methods and RMSE values.

Algorithm	Optimized Feature Set	RMSE
GA	VEC, δG, δr, γ, ΔHmix, ΔGmix, F, G	0.4133
RFE	Tm, VEC, e/a, δG, δr, γ, ΔHmix, ΔSmix, ΔGmix, F, w, μ, Λ, Ω, E, G	0.4137
SFS	Tm, VEC, δG, δr, γ, ΔHmix, ΔSmix, ΔGmix, Δχ, A, F, w, μ, Λ, Ω, η	0.4145
SBS	δG, Λ, Ω, G	0.4135
Ridge	δG, ΔHmix, VEC, δr, F, Λ, Ω	0.4139

## Data Availability

The original contributions presented in this study are included in the article. Further inquiries can be directed to the corresponding author.
